# Integrated Proteomic and Metabolomic Profiling of *Phytophthora cinnamomi* Attack on Sweet Chestnut (*Castanea sativa*) Reveals Distinct Molecular Reprogramming Proximal to the Infection Site and Away from It

**DOI:** 10.3390/ijms21228525

**Published:** 2020-11-12

**Authors:** Iñigo Saiz-Fernández, Ivan Milenković, Miroslav Berka, Martin Černý, Michal Tomšovský, Břetislav Brzobohatý, Pavel Kerchev

**Affiliations:** 1 Phytophthora Research Centre, Department of Molecular Biology and Radiobiology, Faculty of AgriSciences, Mendel University in Brno, Zemědělská 3, 61300 Brno, Czech Republic; saizfern@mendelu.cz (I.S.-F.); miroslavberka94@gmail.com (M.B.); martincerny83@gmail.com (M.Č.); brzoboha@ibp.cz (B.B.); 2 Phytophthora Research Centre, Department of Forest Protection and Wildlife Management, Faculty of Forestry and Wood Technology, Mendel University in Brno, Zemědělská 3, 61300 Brno, Czech Republic or ivan.milenkovic@sfb.bg.ac.rs (I.M.); michal.tomsovsky@mendelu.cz (M.T.); 3 Forest Protection, Department of Forestry, Faculty of Forestry, University of Belgrade, Kneza Višeslava 1, 11030 Belgrade, Serbia

**Keywords:** sweet chestnut, *Phytophthora cinnamomi*, proteomics, metabolomics

## Abstract

*Phytophthora cinnamomi* is one of the most invasive tree pathogens that devastates wild and cultivated forests. Due to its wide host range, knowledge of the infection process at the molecular level is lacking for most of its tree hosts. To expand the repertoire of studied *Phytophthora*–woody plant interactions and identify molecular mechanisms that can facilitate discovery of novel ways to control its spread and damaging effects, we focused on the interaction between *P. cinnamomi* and sweet chestnut (*Castanea sativa*), an economically important tree for the wood processing industry. By using a combination of proteomics, metabolomics, and targeted hormonal analysis, we mapped the effects of *P. cinnamomi* attack on stem tissues immediately bordering the infection site and away from it. *P. cinnamomi* led to a massive reprogramming of the chestnut proteome and accumulation of the stress-related hormones salicylic acid (SA) and jasmonic acid (JA), indicating that stem inoculation can be used as an easily accessible model system to identify novel molecular players in *P. cinnamomi* pathogenicity.

## 1. Introduction

Sweet chestnut (*Castanea sativa* Mill.) is one of the most ecologically and economically important tree species from the *Fagaceae* family that is autochthonous in Southern Europe and in Asia Minor. It is widely grown in planted and coppice forests for the wood processing industry, for ecological protection purposes, as amenity and ornamental tree, and for nut production [1,2,3]. Sweet chestnut is endangered by two major disease threats, chestnut blight and ink disease. Ink disease is caused by the pathogens from the *Phytophthora* genus that are fungi-like organisms within the SAR supergroup, inflicting significant economic and ecological losses in chestnut forests [4]. Two *Phytophthora* species are known as the main causes of ink disease on sweet chestnut, namely *P. cinnamomi* Rands and *P. ×cambivora* (Petri) Buisman [5]. Both *P. cinnamomi* and *P. ×cambivora* are cosmopolites and polyphagous pathogens that attack numerous woody plants and other host species, causing significant losses worldwide [5,6,7,8]. *P. cinnamomi*, in particular, represents one of the most aggressive and notorious plant pathogens, which has been responsible for numerous devastating epidemics [5,7,9]. *P. cinnamomi* has been mainly associated with ink disease in Western Europe, while *P. ×cambivora* is the main cause of ink disease in Central and South-eastern Europe [5,10]. However, *P. cinnamomi* has also been found infecting sweet chestnut in Greece [2], suggesting that its spread may have been vastly underestimated, and that it is most likely already co-occurring together with *P. ×cambivora* and slowly spreading to the other chestnut stands in Europe. Although low temperatures are a limiting factor for *P. cinnamomi* spreading into European forests [5], recent findings in alpine environments and its ability to sporulate at low temperatures in vitro [11] suggests that colder areas of Central and Northern Europe may be at serious risk of infection. This is of particular importance in the light of evident climate changes and projected future spread of *P. cinnamomi* [7].

Infection of chestnut trees with *P. cinnamomi* predominantly occurs through the roots, leading to root deterioration. *P. cinnamomi* is a hemibiotrophic oomycete which upon colonization establishes a biotrophic interaction with its host. During the biotrophic phase, the pathogen develops haustoria that facilitate reabsorption of nutrients from the host and delivery of effector proteins and small molecules that manipulate host defense [12]. Once the hyphae have spread throughout the endodermis and vascular tissue, necrotrophy can be observed [13,14]. Infected trees display various disease symptoms like leaf atrophy, chlorosis, wilting, flag-like appearance of wilted leaves, and tree dieback [5,10]. From the infected roots, the pathogen usually spreads to the collar, bark, and cambial tissues of the stems, resulting in flame-shaped bleeding cankers and lesions [5,10]. Despite being mainly a root pathogen, under certain conditions *P. cinnamomi* can also infect trunks directly via lenticels, adventitious roots, or wounds [15]. Stem inoculation has been successfully employed to determine the proteomic, metabolomic, and transcriptomic alterations caused by *Phytophthora* attack on several tree species [16,17,18]. Infection rates tend to be highest in spring when maximal cambial activity coincides with the peak in pathogen’s sporulation [19]. Infected bark cells display altered shapes and plugged plasmodesmata [20]. The infection process is accompanied with callose, starch, and crystal deposition, and the establishment of a new periderm that limits secondary infection [15]. The newly formed periderm often provides only temporary protection which, once breached, allows invasion of phloem and xylem tissues. Once the xylem has been invaded, the plant faces the possibility of xylem disfunction, resulting in hydraulic failure and sudden tree death [15]. Interestingly, leaf photosynthetic rates tend to start decreasing prior to xylem failure. This decrease has been suggested to be a consequence of phloem tissue destruction (girdling), which impairs plant carbon allocation and causes starch to accumulate in the leaves, ultimately leading to photoinhibition [21].

Plants employ a diverse array of constitutive and inducible defense mechanisms that are being deployed during pathogen attack. Physical and chemical barriers, such as waxy layers and antimicrobial compounds, are often the first line of defense against invading pathogens [22]. Upon recognition of pathogen-associated molecular patterns (PAMPs), plants mount PAMP-triggered immunity (PTI). PTI is accompanied by the generation of reactive oxygen species (ROS), activation of protein kinase cascades, reinforcement of physical barriers, and production of numerous defense-related molecules [23]. Plant pathogens employ secreted effector proteins that interfere with plant defense mechanisms and facilitate infection [24]. Detection of pathogen effectors by the products of disease-resistant R genes activates effector-triggered immunity (ETI). Pathogen effectors can be delivered to the apoplast (e.g., cell wall degrading enzymes and elicitins) or cytosol where they target various intracellular processes [25]. Cytosolic uptake is a key part of the infection process and many oomycete effector proteins possess N-terminal RxLR or LxLFLAK motifs that facilitate their uptake [23]. Additionally, plant proteins involved in exocytotic vesicle trafficking, such as v-SNARE or the VAMP72 family proteins, are found in the pathogenic membrane formation interfaces and contribute to plant defense by delivering anti-microbial proteins [26,27]. Plant membrane trafficking can be at the same time targeted by *Phytophthora* effectors in order to enhance their virulence [28,29,30]. Plant hormonal signaling pathways are commonly targeted by *Phytophthora* effector proteins due to their crucial roles in plant defense [15]. Moreover, filamentous plant pathogens can synthesize phytohormones and their derivatives that can manipulate and hijack the hormonal homeostasis of their plant hosts [31]. 

Here, we explored the molecular mechanisms associated with *P. cinnamomi* attack on chestnut. Stem infection was used as an easily accessible model system to probe the proteomic and metabolic response to *P. cinnamomi*. Our analysis revealed a significant molecular impact along the infected stem and indicated proteomic and metabolic alterations in the leaves of infected saplings. Taken together, the reported results point towards mobilization of SA and JA signaling pathways, which likely reflects the hemibiotrophic lifestyle of *P. cinnamomi*.

## 2. Results

### 2.1. Stem Inoculation as a Model System to Study Phytophthora cinnamomi Infection on Chestnut

**Figure 1:**
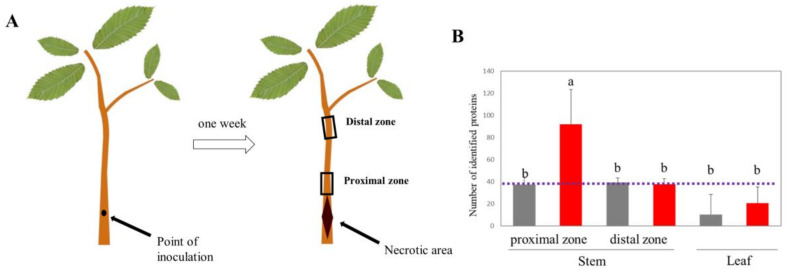
Stem inoculation as a model system to study *Phytophthora cinnamomi* infection on chestnut. (**A**) A schematic depiction of the experimental design. Two-year-old sweet chestnut saplings were stem wounded and subsequently inoculated with mycelium from *P. cinnamomi* mating type A2. Control plants received mock treatment. One week later, stem sections bordering the necrotic area (proximal zone) and around 20 cm upstream of it (distal zone) were harvested and used to extract total proteins and metabolites. (**B**) Number of high-scoring proteins assigned to the *P. cinnamomi* proteome in inoculated (red) and mock-treated (gray) chestnut saplings. Error bars represent standard deviation of at least three biological replicates. A false positive threshold corresponding to the maximal number of identified *Phytophthora-*like proteins in the mock-treated tissues is depicted by a dashed line. Different letters represent statistically significant differences according to Kruskal–Wallis test (*p* < 0.05).

To sidestep the limitations associated with inoculation and sampling of infected root tissues and explore alternative experimental model systems to study the interaction between *P. cinnamomi* and chestnut, we inoculated stems of two-year-old sweet chestnut saplings with *P. cinnamomi* (Figure 1A). Seven days after inoculation, the tissue proximal to the inoculation site displayed necrotic lesions, indicating active *P. cinnamomi* colonization. The presence of the pathogen in the living stem bordering the necrotic tissue was confirmed by extracting proteins and querying the measured peptide spectra against the *P. cinnamomi* proteome. Fourteen proteins unique for *P. cinnamomi*, such as transcriptional regulator NmrA, HIT family protein, putative secretory protein of the CAP family, and carbohydrate-active enzymes (CAZymes) were identified (Figure 1B). The rest of the identified protein families (291) originated from evolutionarily conserved sequences shared between *P. cinnamomi* and chestnut. No unique *P. cinnamomi* proteins were found in stem sections upstream of the inoculation site and in the leaves of infected plants. Taken together, the development of necrotic lesions upon *P. cinnamomi* inoculation and the presence of *P. cinnamomi* proteins in the immediate vicinity of the infection site but not away from it indicated active colonization and supported the use of chestnut stem inoculation as a viable model system to study *P. cinnamomi*–chestnut interaction at the molecular level.

### 2.2. Phytophthora cinnamomi Infection Triggers Distinct Proteome Reprogramming Proximal to the Infection Site and Away from It

**Figure 2:**
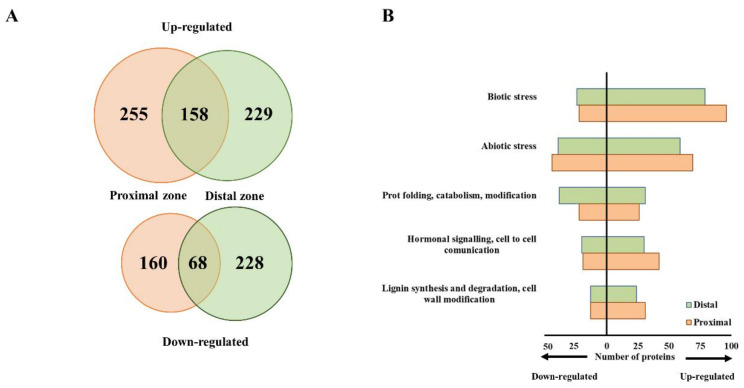
Proteome rearrangement in chestnut stems infected with *Phytophthora cinnamomi*. (**A**) Venn diagrams depicting the overlap between proteins that accumulated (upper panel) or decreased (lower panel) in stem sections proximal and distal to the infection zone in comparison to mock-treated controls (∣FC∣ ≥ 2, *p* ≤ 0.05). (**B**) Top five most represented functional categories of differentially abundant proteins in proximal and distal stem sections upon *P. cinnamomi* attack. Bars represent number of accumulated (right side) or decreased (left side) proteins. Proteins were assigned to each functional category based on their putative *Arabidopsis* orthologs identified using the Uniprot database.

To get an insight into the molecular mechanisms that accompany the *P. cinnamomi* attack on chestnut, we assessed the global proteome changes in inoculated stems. To achieve a spatial resolution in understanding the infection process, we collected samples immediately bordering the necrotic site and approximately 20 cm upstream of it. The chestnut (*Castanea sativa*) genome had not been sequenced yet, so we used the genomes of the closely related *Castanea* species, *C. dentata* and *C. mollissima*, for peptide sequence annotation and protein identification [32,33]. Four hundred and thirteen proteins accumulated in the stem section proximal to the infection site, whereas the abundances of 228 proteins decreased upon *P. cinnamomi* infection according to a cut-off of ∣FC∣ ≥ 2 and *p* ≤ 0.05 (Figure 2A). A similar number of proteins were differentially regulated (387 up and 296 down) distantly from the infection site.

**Figure 3:**
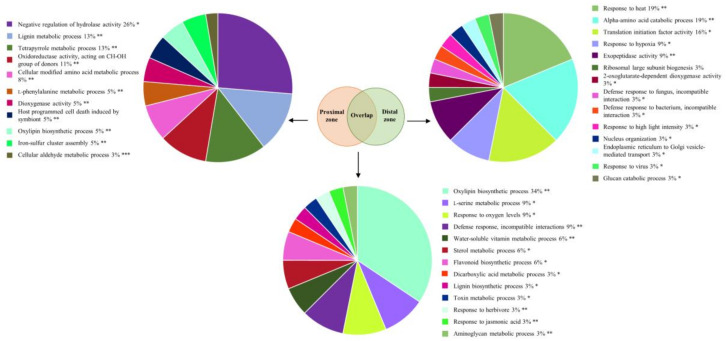
Functional annotation enrichment of proteins that accumulated in chestnut stem after *Phytophthora cinnamomi* infection. Pie charts depict functional categories assigned by the Cytoscape ClueGO plug-in using *Arabidopsis* orthologs of chestnut proteins that accumulated exclusively in stem sections proximal (left panel) and distal (right panel) to the infection site or were similarly induced in both sections (lower panel). Numbers indicate percentage of terms per group and asterisks indicate group *p*-value (* *p* < 0.05; ** *p* < 0.01; *** *p* < 0.005).

To make use of the available bioinformatics tools for functional enrichment, none of which contained *Castanea*-specific information, we queried the obtained protein sequences against the *Arabidopsis thaliana* proteome in order to identify orthologous proteins that were used for downstream analysis (Table S1). The majority of differentially abundant proteins isolated from both the proximal and distal stem sections were associated with biotic stress responses, followed by abiotic stress (Figure 2B). Their numbers were higher in the proximal zone, indicating a more pronounced response to *P. cinnamomi* attack. Similarly, more proteins associated with hormonal signaling and cell wall modification accumulated in the zone neighboring the necrotic area. Functional enrichment analysis of the proteins that uniquely accumulated in the stem section proximal to the infection site identified, among others, lignin metabolic process, l-phenylalanine metabolic process, and host programmed cell death (Figure 3). Proteins orthologous to cinnamyl alcohol dehydrogenase (CAD) 7 and 9 from *Arabidopsis*, for example, were specifically induced upon infection only in the proximal zone. CADs are important players in structural lignification during devolvement but are also strongly induced upon pathogen attack, leading to localized lignification [34]. Interestingly, CAD9 has been shown to be a target of multiple *Phytophthora* Avr3a-like effectors [35]. An ortholog of the *Arabidopsis* senescence marker SENESCENCE-ASSOCIATED GENE 21 (SAG21) also accumulated exclusively in the stem section bordering the infection zone. Two proteins orthologous to the *Arabidopsis* phenylalanine biosynthetic enzymes, arogenate dehydratase 6 and phenylalanine ammonia-lyase 2 (PAL2), were also induced upon infection only in the proximal zone. One hundred and fifty-eight proteins were commonly induced in the stem sections proximal and distal to the necrotic area (Figure 3). Among the functional categories that were overrepresented in this protein list were oxylipin biosynthetic process, defense response during incompatible interaction, lignin biosynthetic process, and response to jasmonic acid (JA). Moreover, a protein orthologous to the *Arabidopsis* 1-aminocyclopropane-1-carboxylic acid oxidase (ACO), involved in ethylene (ET) biosynthesis, accumulated in both stem sections. Similarly, an ortholog of PATHOGENESIS-RELATED 1 (PR1), a widely accepted molecular marker for salicylic acid (SA)-mediated signaling, was induced proximally and distally from the infection site implying that the presence of *P. cinnamomi* triggers partially overlapping defense responses in both stem sections. The top four overrepresented functional categories assigned to the proteins exclusively induced in the distal stem section were response to heat, amino acid catabolic process, translational initiation factor activity, and response to hypoxia (Figure 3).

**Figure 4:**
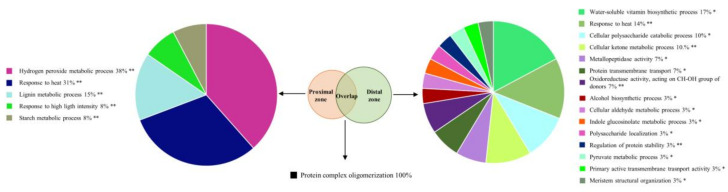
Functional annotation enrichment of proteins that decreased in chestnut stem after *Phytophthora cinnamomi* infection. Pie charts depict functional categories assigned by the Cytoscape ClueGO plug-in using *Arabidopsis* orthologs of chestnut proteins that decreased exclusively in stem sections proximal (left panel) and distal (right panel) to the infection site or similarly decreased in both sections (lower panel). Numbers indicate percentage of terms per group and asterisks indicate group *p*-value (* *p* < 0.05; ** *p* < 0.01).

The most overrepresented functional category of all proteins that decreased exclusively in stem sections proximal to the infection site was hydrogen peroxide metabolic process (Figure 4). It contained, among others, proteins orthologous to the *Arabidopsis* peroxidase 53 and peroxidase superfamily protein. Starch metabolism was similarly overrepresented with glucose-1-phosphate adenylyltransferase and beta-glucosidase 17 and 44 being decreased uniquely in the proximity of the infection site. Two hundred and twenty-eight proteins exclusively decreased in stem sections distant from the infection site. Among the functional categories that were overrepresented in this protein list were the polysaccharide metabolic process and polysaccharide localization (Figure 4). In particular, proteins orthologous to *Arabidopsis* granule-bound starch synthase 1, isoamylase 3, and alpha amylase-like protein were identified. Interestingly, response to heat was the second most overrepresented category in stem sections both proximal and distal to the infection site, but the specific proteins, which included mainly members of the chaperone and heat-shock protein (HSP) families, were largely different in each stem section.

A number of proteins showed a contrasting response in the stem sections proximal and distal to *P. cinnamomi* infection (Table 1). For example, an ortholog of the *Arabidopsis* universal stress protein PHOS32-like, which in *Arabidopsis* is phosphorylated after *P. infestans* elicitation [36], accumulated in the vicinity of the necrotic area, but its abundance decreased away from it. A similar expression pattern was observed for a protein orthologous to *Arabidopsis* SENSESCENCE-RELATED GENE 1 (SRG1)-like, which is highly induced during senescence [37]. Two vesicle transport-related proteins orthologous to *Arabidopsis* VESICLE TRANSPORT V-SNARE 13-LIKE and VESICLE-ASSOCIATED PROTEIN 2-1 also increased close to the infection site but decreased in the stem section distal to it. Proteins involved in exocytotic vesicle trafficking are often targeted by *Phytophthora* effectors and appear to be susceptibility factors [38]. Taken together, our results reveal a significant rearrangement of the proteome in both proximal and distal stem sections following *P. cinnamomi* infection. The responses of both tissue types shared similarities, but at the same time displayed distinct differences reflecting their spatial position in relation to the infection.

### 2.3. The Stress-Related Amino Acid Proline Accumulates upon Phytophthora cinnamomi Infection

**Figure 5:**
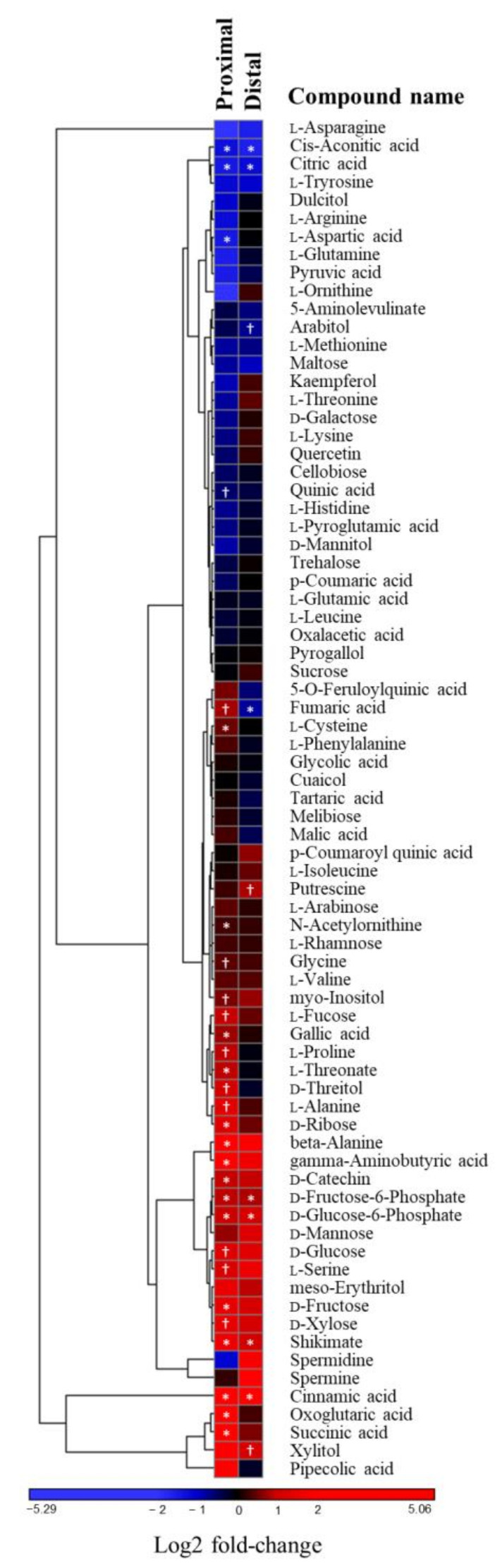
Effect of *Phytophthora cinnamomi* attack on chestnut metabolism. Heatmap showing changes in metabolites isolated from chestnut stem sections proximal and distal to the necrotic area seven days after *P. cinnamomi* inoculation relative to mock-treated controls. Symbols indicate statistically significant differences according to Student’s *t*-test (*p* < 0.1 † and *p* < 0.05 *).

The significant impact of the *P. cinnamomi* attack on the plant proteome prompted us to investigate how the infection process is reflected at the metabolite level. To this end, we quantified the steady-state levels of primary polar metabolites (mainly amino acids, sugars, and organic acids) in stem sections proximal and distal to the necrotic area resulting from *P. cinnamomi* infection. Most of the identified metabolites were either not affected by the presence of *P. cinnamomi* or responded similarly in both proximal and distal stem sections (Figure 5, Table S2). Among the metabolites that displayed a differential response upon infection were the stress-related amino acid proline and ɣ-aminobutyric acid (GABA) which modulate plant tolerance to abiotic and biotic stresses [39,40]. Both of them accumulated exclusively in the stem section proximal to the infection. A similar distribution pattern was observed for gallic acid. In contrast, the content of the amino aspartate, which is among the major nitrogen storage forms, was more negatively affected in the vicinity of the necrotic site. Accumulation of shikimic and cinnamic acid was detected in stem sections both proximal and distal to the infection site. The flavonoid d-catechin, on the other hand, accumulated close to the infection site only.

### 2.4. Phytophthora cinnamomi Attack is Accompanied with Perturbation of Plant Hormonal Homeostasis

**Figure 6:**
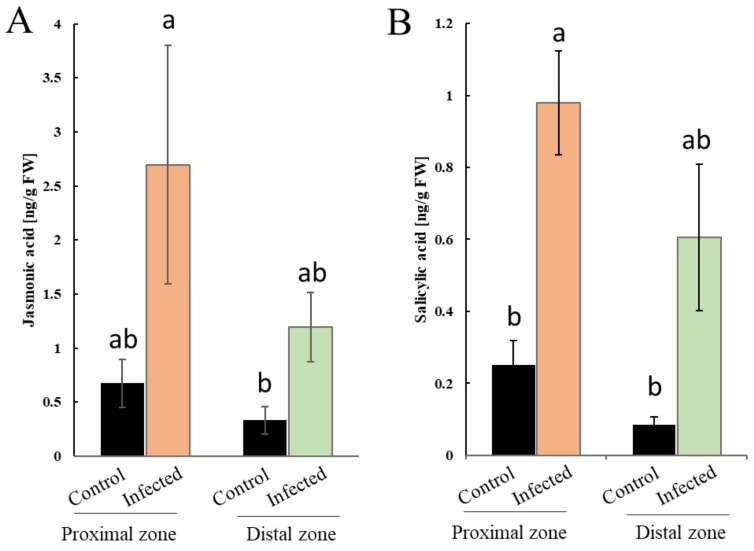
Effect of *P. cinnamomi* infection on plant hormonal levels. (**A**) Jasmonic acid content in stem sections proximal (orange) and distal (green) to the necrotic area seven days after *P. cinnamomi* inoculation in comparison to mock-treated controls (black). (**B**) Salicylic acid content in stem sections proximal (orange) and distal (green) to the necrotic area seven days after *P. cinnamomi* inoculation in comparison to mock-treated controls (black). Bars represent averages of four biological replicates ± SE. Different letters depict statistical differences according to one-way analysis of variance (ANOVA) with Tukey’s post hoc test (*p* < 0.05).

Defense responses against pathogens are governed by hormonal signaling pathways with induction of SA signaling being associated predominantly with biotrophic interactions, whereas JA-related signal transduction pathways are mainly involved in necrotrophic interactions and insect infestation [41]. To assess the impact of *P. cinnamomi* on plant hormonal levels, we quantified SA and JA content in stem sections proximal and distal to the infection site. *P. cinnamomi* infection significantly increased SA levels in both stem sections (Figure 6A). Similarly, enhanced JA levels were observed both locally and distally in relation to the necrotic zone (Figure 6B). These results indicate that *P. cinnamomi* infection has a significant impact on plant hormonal homeostasis which likely modulates stress signaling pathways.

### 2.5. Phenylpropanoid Biosynthesis Pathway is Enriched following P. Cinnamomi Infection

**Figure 7:**
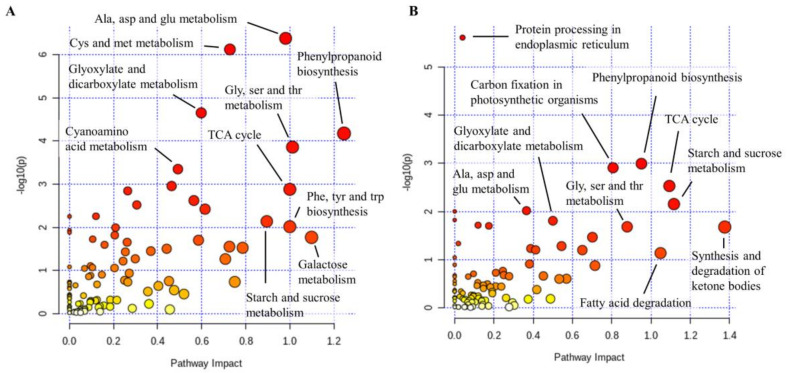
Functional enrichment and pathway topology analysis of proteins and metabolites affected in chestnut stem upon *Phytophthora cinnamomi* stem inoculation. (**A**) Integrated metabolic pathway analysis of proteins and metabolites differentially regulated in stem sections proximal to the necrotic zone seven days after *P. cinnamomi* inoculation performed using the “Joint Pathway Analysis” tool implemented in MetaboAnalyst 4.0. (**B**) Integrated metabolic pathway analysis of proteins and metabolites differentially regulated in stem sections distal to the necrotic zone seven days after *P. cinnamomi* inoculation performed using the “Joint Pathway Analysis” tool implemented in MetaboAnalyst 4.0.

To pinpoint with higher evidence the pathways underlying *P. cinnamomi* pathogenicity on chestnut, we mapped proteins significantly altered upon infection together with significant metabolites to metabolic pathways for functional enrichment and pathway topology analysis using the “Joint Pathway Analysis” tool implemented in MetaboAnalyst 4.0 [42]. The pathways with the highest impact in the stem section proximal to the infection site were phenylpropanoid biosynthesis; phenylalanine, tyrosine, and tryptophan biosynthesis; galactose metabolism; alanine, aspartate, and glutamate metabolism; and TCA cycle (Figure 7A). Phenylpropanoid biosynthesis had a similar high impact upstream from the infection site together with synthesis and degradation of ketone bodies, starch, and sucrose metabolism, and TCA cycle (Figure 7B). Among the enzymes from the phenylpropanoid biosynthesis, a protein orthologous to *Arabidopsis* caffeoyl-CoA O-methyltransferase 1 accumulated in stem sections proximal to the inoculation site, whereas orthologs of feruloyl CoA ortho-hydroxylase 1 and caffeic acid 3-O-methyltrasnferase 1 accumulated both in the vicinity of the inoculation site and upstream of it.

### 2.6. P. Cinnamomi Infection Alters the Chestnut Leaf Proteome and Metabolome

**Figure 8:**
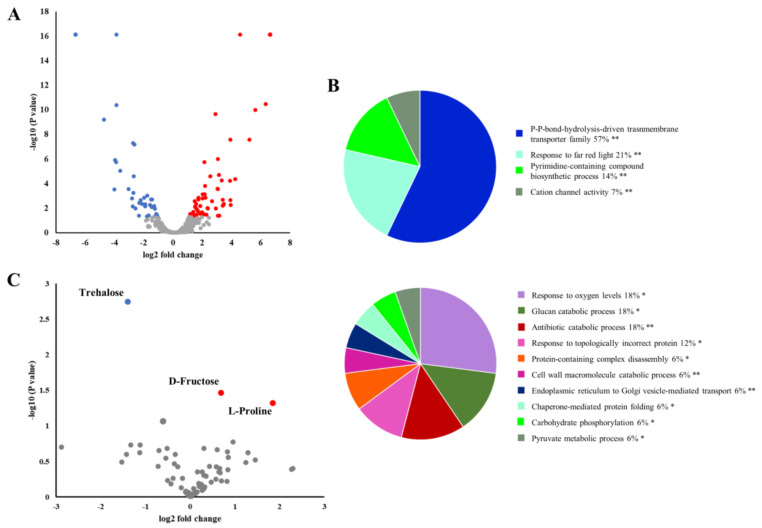
Effect of *Phytophthora cinnamomi* stem infection on chestnut leaf proteome and metabolome. (**A**) Volcano plot of proteins upregulated (red) and downregulated (blue) in chestnut leaves seven days after stem inoculation with *P. cinnamomi* (log2 FC ≥ 1 and *p* ≤ 0.05). (**B**) Functional enrichment analysis based on *Arabidopsis* orthologs of chestnut proteins that accumulated (upper panel) or decreased (lower panel) in leaves of *P. cinnamomi*-inoculated chestnut plants after seven days performed using Cytoscape ClueGO plug-in. Numbers indicate percentage of terms per group and asterisks indicate group *p*-value (* *p* < 0.05; ** *p* < 0.01). (**C**) Volcano plot of the leaf metabolites with increased (red) and decreased (blue) concentration seven days after stem inoculation with *P. cinnamomi* (*p* value ≤ 0.05).

Plants utilize long-distance signaling cascades to transmit information about pathogen attack and prepare distant tissues for subsequent invasion and/or alter their whole plant physiology that can ultimately affect their response to a broader range of biotic or abiotic stresses [43]. To assess whether information about *P. cinnamomi* infection is relayed to distant plant tissues, we probed the leaf proteomic and metabolomic profiles of infected chestnut saplings. One hundred and fifty-four proteins accumulated, whereas the abundance of 194 proteins decreased in comparison to leaves collected from mock-treated saplings (∣FC∣ ≥ 2 and *p* ≤ 0.05; Figure 8A; Table S1). In contrast to the many proteins that could be attributed to biotic and abiotic stress responses found in the infected stems, no stress-related functional categories were observed in the leaves (Figure 8B).

Similarly, the metabolomic profile of leaves from infected saplings was markedly different from that of stem sections proximal and distal to the necrotic zone. A limited number of metabolites accumulated (2) or decreased (1) following infection (Figure 8C, Table S2). Despite the limited overlap between the metabolic changes in leaves and stem sections, proline increase was also observed in leaves of infected saplings. Taken together, these results indicate that *P. cinnamomi* infection also impacts the leaf proteome and metabolome.

## 3. Discussion

In this study, we characterized the susceptible interaction between the invasive oomycete *Phytophthora cinnamomi* and one of its economically important hosts *Castanea sativa*. By integrating proteomic and metabolomic profiling and targeted hormonal analysis, we revealed a major molecular impact of *P. cinnamomi* attack on sweet chestnut.

### 3.1. P. Cinnamomi Inoculation Triggers a Defense Response Common to Abiotic Stress

*P. cinnamomi* is a soil-borne pathogen that causes root rot (ink disease) and eventually tree death [5,10]. However, despite being mainly a root pathogen, *P. cinnamomi* can also infect trunks and stems directly. Stem inoculation is accompanied with phloem necrosis and xylem blocking that ultimately leads to perturbation of plant water relations [44]. As a result, the response against *Phytophthora* shares many similarities with drought stress and leads to negative effects on photosynthesis and photooxidative damage. In fact, eucalyptus trees display a marked water stress-response following stem inoculation with *P. cinnamomi* [17]. Numerous proteins implicated in abiotic stress responses accumulated in the stem of infected plants, supporting the idea that the *P. cinnamomi* attack negatively perturbs plant physiology. Moreover, we observed accumulation of proline in stem sections bordering the infection zone and in leaves of infected saplings, indicating altered water potential that is likely due to blocked vasculature. The role of proline during stress responses has been attributed to its osmolite properties and antioxidant characteristics. Proline accumulation has also been shown to modulate programmed cell death during plant–pathogen interactions, implying that its role upon *P. cinnamomi* attack might extend beyond its osmolite properties [45].

### 3.2. Manipulation of Plant Defense by P. Cinnamomi

Several heat-shock proteins (HSPs) which normally accumulate under diverse abiotic stress conditions [46] were repressed following *P. cinnamomi* infection in the stem sections proximal and distal to the necrotic zone. Apart from their role in abiotic stress, HSPs are important players in defense signaling during pathogen attacks by modulating the stability and accumulation of resistance proteins [47]. Silencing of small HSPs has been linked to a decrease in the expression of defense-related genes, such as PR1 and PR4 in tobacco, and the perturbation of SA signaling [48]. Interestingly, HSPs are prime targets for effector proteins from *Phytophthora* which leads to increased pathogen virulence [49,50]. Further confirming the important role of HSPs in response to pathogens is the fact that *Phytophthora* species have been shown to decrease the levels of host HSPs by targeting their promoters [44].

### 3.3. SA-Mediated Defense Signaling Pathways Are Activated upon P. Cinnamomi Attack

SA signaling plays a crucial role in the defense against biotrophic pathogens and establishment of a hypersensitive response (HR). The importance of SA signaling is not limited to plant–biotroph interactions, but has been also reported in defense against sucking insects and hemibiotrophs [51]. Contrasting evidence exists about the role of SA signaling during *Phytophthora* infection. Whereas SA-pretreatment on rubber tree reduced the disease symptoms against *P. palmivora* [52], *P. cinnamomi* attack on chestnut did not alter SA levels [53]. Interestingly, we detected increased SA levels in stem sections both proximal and distal to the infection (Figure 6B), indicating that *P. cinnamomi* mounts a defense response similar to that triggered by biotrophic pathogens. Further corroborating this observation was the accumulation of proteins from the phenylalanine ammonia-lyase (PAL) pathway and cinnamic acid, an intermediate in the PAL pathway. SA can be synthesized via the isochorismate synthase and PAL pathway, and the importance of both pathways can vary between species and conditions [54]. Activation of SA signaling upon *Phytophthora* infection is believed to modulate H_2_O_2_ content and antioxidant enzyme levels, and impact lignin deposition [52,55,56].

Induction of SA synthesis during pathogen attack has been correlated with upregulation of the flavan-3-ol pathway, and particularly with increases in catechin levels [57]. Flavan-3-ols are part of the constitutive and induced chemical defense against fungal pathogens in woody plants that could inhibit spore germination and prevent hyphal growth [58,59]. The accumulation of d-catechin observed in *Phytophthora*-infected chestnut stems, together with the increase of gallic acid required for biosynthesis of their gallate conjugates, indicates that similar defense mechanisms can be deployed against oomycetes.

### 3.4. Activation of JA Signaling upon P. Cinnamomi Attack Likely Plays Multiple Roles during Infection

JA signaling fuels the accumulation of diverse defense compounds in various plant species, including *Castanea*, and promotes resistance to necrotrophic pathogens and chewing insects [60,61]. JA and its biologically active conjugate JA-Ile have been described to increase in *P. cinnamomi*-infected roots of both susceptible and resistant *Castanea* clones [53]. We observed accumulation of several JA biosynthetic enzymes, such as allene oxide synthase and linoleate 13S-lipoxygenase, which was accompanied with increased JA content in stem sections proximal and distal to the infection zone (Figure 6). Moreover, other linolenic acid-derived oxylipins have been reported to play a direct role in oomycete infection by displaying antifungal activities and have been proposed to be important components of plant defense against *Phytophthora* [62,63]. Proteins related to the oxylipin biosynthetic process were overrepresented in chestnut stems infected with *P. cinnamomi* (Figure 3). Taken together, our results indicate that activation of JA signaling accompanies *P. cinnamomi* attack on chestnut.

JA signaling has been shown to modulate sugar metabolism during the interaction between soybean and *P. sojae* via increase in extracellular invertases activity [64]. We observed accumulation of several sugars including glucose and fructose in *Phytophthora*-infected chestnut stems (Figure 5). Sugars are involved in plant defense as substrates for several secondary metabolites, including the building blocks of lignin [65]. Furthermore, soluble hexoses have been shown to activate genes related to the shikimate pathway [66]. The accumulation of sugars close to the infection site, however, is not always beneficial, as the pathogen can use them for its own benefit [67]. Moreover, some pathogens have developed mechanisms to hijack host carbohydrate metabolism [67,68].

### 3.5. Establishment of Physical Barriers is a Key Part of the Defense against P. Cinnamomi

The phenylpropanoid pathway is a prominent feature of plant defense against pathogens, which tends to be upregulated in resistant lines [69]. This pathway generates precursors for the localized biosynthesis of lignin and suberin, which can be used to establish physical barriers and prevent pathogen spreading [70]. Shikimic and cinnamic acid, precursors of the phenylpropanoid biosynthetic pathway, accumulated in chestnut stems after *P. cinnamomi* infection (Figure 5). Additionally, several proteins related to phenylpropanoid and lignin biosynthesis, such as peroxidases, S-adenosylmethionine synthases, and CADs, were upregulated in both stem sections (Figure 2B). Phenylpropanoids also exhibit a broad-spectrum antimicrobial activity and have been associated with plant hypersensitive response [70]. Furthermore, CADs could also contribute to the defense against *P. cinnamomi* by reducing *Phytophthora* aldehyde aromatic effectors into alcohols, and thus inactivating them [71].

Flavonoids have been suggested to promote callus and tylose formation and closure of vascular tissue to prevent pathogen expansion [72]. Additionally, flavonoid glycosides can form a hard and crystalline structure that blocks the path of the pathogen and cannot be broken by cell wall degrading enzymes [73]. Flavonoids are synthesized via the phenylpropanoid pathway which was overrepresented in infected stems. Specifically, we observed accumulation of proteins orthologous to *Arabidopsis* feruloyl CoA ortho-hydroxylase 1 and flavone 3’-O-methyltransferase 1 next to the infection site, implying that flavonoids can play a role in the interaction between *Phytophthora* and chestnut.

Proteins involved in vesicle trafficking seem to be key susceptibility factors during early pathogen establishment [38]. Interestingly, we observed accumulation of V-SNARE and VAMP family proteins close to the infection site, but decreasing concentrations upstream of it (Table 1). Proteins from those families have been suggested to mediate pre-invasion resistance, ETI response, and participate in the secretion of anti-microbial proteins [26,27]. They are also thought to be involved in post-invasive defense by carrying resistance proteins to the extrahaustorial membrane [74]. We also observed accumulation of proteins secreted into the extracellular space, such as osmotins (OSMOTIN 34 and OSMOTIN-LIKE proteins), endochitinases (basic endochitinase B and endochitinase EP), and pectinesterases (PECTINESTERASE 2-LIKE). Osmotins can inhibit hyphal growth by increasing the permeability of the pathogen membrane and thus impairing their ability to maintain a pH gradient [75], while endochitinases and pectinesterases are cell wall degrading enzymes that can damage the pathogen without harming the host [76,77]. Additionally, pectinesterases are also involved in the reinforcement of a host’s own cell wall by calcium–pectate gel apposition, which has been associated with resistance to several *Phytophthora* species [78].

### 3.6. Increase of Sugar Content in Chestnut Leaves after P. Cinnamomi Inoculation

The molecular response observed in leaves of stem-infected chestnut saplings was markedly different to that observed in the stem and did not display a clear stress response signature. A marked exception was the accumulation of proline which could be attributed to the reduction in water uptake as a result of vasculature destruction during *Phytophthora* stem infection. Impaired phloem transport has also been linked to the accumulation of carbohydrates and starch in leaves of infected plants that could ultimately lead to photoinhibition of photosynthesis [15]. We detected accumulation of d-fructose, together with a decrease of several proteins related to starch degradation (Alpha-glucan phosphorylase, isoamylase 3, phosphoglycan, and water dikinase) suggesting that the *P. cinnamomi* attack on chestnut could also impact leaf sugar and starch levels.

## 4. Materials and Methods

### 4.1. Plant Material

Sweet chestnut (*Castanea sativa*) saplings were grown from seeds collected in a natural sweet chestnut forest in Serbia (42°33′52″ N; 21°50′58″ E). Seeds were sown in 20 l plastic boxes, containing an autoclaved mixture of peat, sand, and perlite (1/1/1) and grown under controlled conditions at 22–25 °C. Plants were watered twice per week until the soil reached field capacity.

### 4.2. P. Cinnamomi Inoculation

A *Phytophthora cinnamomi* isolate (TJ349) belonging to A2 mating type was selected from the Phytophthora Research Centre (www.phytophthora.org/) culture collection and developed on V8A media (prepared with 100 mL/l of clarified V8 juice (Pfanner, Lauterach, Austria), 20 g/l of agar (Sigma Aldrich, St. Louis, MO, USA), 2 g/l of CaCO_3_, and 900 mL/l of distilled water), and incubated for 5 days at 20 °C in the dark. Inoculation of stems of two-year-old plants was performed using the under-bark stem inoculation test. Wounds were made using a metal cork borer of 6 mm in diameter at 5–7 cm from the ground level, sterilized in 70% alcohol, and briefly burned over an open flame. From the growing edge of five-day-old colonies, pieces of agar with mycelium were taken with the same metal cork borer and plated with the mycelium side to exposed wood. Inoculation places were sealed with parafilm and with aluminum foil. Control plants received pieces of sterile agar.

### 4.3. Sample Collection

Stem sections (2 cm in length) were collected right at the end of the necrotic tissue and approximately 20 cm upstream of that area seven days after inoculation. The youngest fully developed leaf of each sapling was also harvested. Samples were flash-frozen in liquid nitrogen, ground to powder in a Retsch MM 400 mill (Retsch GmbH, Haan, Germany), and stored at −80 °C until analysis. Samples from a total of four plants per treatment were collected and processed.

### 4.4. Shotgun Proteomics

Total proteins were extracted as previously described using a combination of phenol/acetone/TCA [79]. Aliquots corresponding to 2.5 μg of peptide were analyzed by nanoflow C18 reverse-phase liquid chromatography using a 15 cm column (Zorbax, Agilent), a Dionex Ultimate 3000 RSLC nano-UPLC system (Thermo Fisher Scientific, Waltham, MA, USA) and the Orbitrap Fusion Lumos Tribrid Mass Spectrometer (Thermo Fisher Scientific) as described previously [80]. The measured spectra were recalibrated and searched against the reference protein database by Proteome Discoverer 2.4, employing Sequest HT (Thermo Fisher Scientific) and MS Amanda 2.0 [81] with the following parameters: Enzyme—trypsin, max two missed cleavage sites; modifications—carbamidomethyl (Cys) and up to three dynamic modifications including met oxidation, Asn/Gln deamidation, N-terminal acetylation; MS1 tolerance—5 ppm (MS Amanda), 10 ppm (Sequest), MS2 tolerance—0.02 Da (MS Amanda), 0.1 Da (Sequest). Only proteins with at least two unique peptides were considered for quantitative analysis. The quantitative differences were determined by Minora, employing precursor ion quantification followed by normalization and background-based *t*-test. The mass spectrometry proteomics data have been deposited to the ProteomeXchange Consortium via the Proteomics Identification Database (PRIDE) [82] partner repository with the dataset identifier PXD022381.

### 4.5. Metabolite Profiling

Polar metabolites were extracted as previously described with few modifications [83]. Samples were derivatized by 20 µL of methoximation solution (40 mg methoxyamine hydrochloride in 1 mL pyridine) and incubated for 90 min at 30 °C with continuous shaking. After the incubation, 80 µL of the silylation solution (N-methyl-N-(trimethylsilyl)trifluoroacetamide) was added and the mixture was incubated for 30 min at 37 °C with continuous shaking. Samples for hormonal analyses were spiked with deuterated salicylic acid (2-hydroxy[^2^ H_4_]benzoic acid; Olchemim, CZ) and processed as described before [84]. Derivatized samples were measured using a Q Exactive GC Orbitrap GC-tandem mass spectrometer and Trace 1300 Gas chromatograph (Thermo Fisher Scientific, Waltham, MA, USA). Samples were injected using the split mode (inlet temperature 250 °C, splitless time 0.8 min, purge flow 5.0 mL/min, split flow 6.0 mL/min) onto a TG-5SILMS GC Column (Thermo Fisher, 30 m × 0.25 mm × 0.25 μm) with helium as a carrier gas at a constant flow of 1.2 mL/min. Metabolites were separated with a 28 min gradient (70 °C for 5 min followed by 9 °C per min gradient to 320 °C and finally 10 min hold time) and ionized using the electron ionization mode (electron energy 70 eV, emission current 50 μA, transfer line and ion source temperature 250 °C). The MS operated in the full scan mode, 60,000 resolution, scan range 50–750 m/z, automatic maximum allowed injection time with automatic gain control set to 1e6, and lock mass (m/z): 207.0323. Data were analyzed by TraceFinder 4.1 with Deconvolution Plugin 1.4 (Thermo) and searched against the NIST2014, GC-Orbitrap Metabolomics library, and in-house library. Only metabolites fulfilling identification criteria (score ≥ 75 and ΔRI < 2%) were included in the final list. The quantitative differences were determined by manual peak assignment in Skyline 20.1 [85], using the extracted ion chromatogram (2 ppm tolerance).

### 4.6. Functional Annotation and Bioinformatics Analysis

The identified protein sequences were queried against the SUBA (https://suba.live/) and NCBI (https://www.ncbi.nlm.nih.gov) databases to generate a list of *Arabidopsis thaliana* and *Quercus suber* homologs, respectively. Putative orthologs with the highest score were selected for downstream data analysis and interpretation. The Uniprot database (https://www.uniprot.org/) was used to assign each of the putative *Arabidopsis* homologs to functional categories. Gene ontology (GO) enrichment analysis was performed using the Cytoscape ClueGO plugin [86]. Functional enrichment and pathway topology analysis was performed using the “Joint Pathway Analysis” tool implemented in MetaboAnalyst 4.0 [42].

## Figures and Tables

**Table 1:** Proteins oppositely regulated upon *Phytophthora cinnamomi* attack in stem sections proximal and distal to the infection zone (∣FC∣ ≥ 2 and *p* ≤ 0.05). Protein identities were assigned based on sequence homology to *Quercus suber* and *Arabidopsis thaliana* proteins.

Protein Annotation	Proximal Zone	Distal Zone
aconitate hydratase, cytoplasmic-like	Up	Down
alpha-amylase-like	Up	Down
aminoacylase-1 isoform X2	Up	Down
beta-glucosidase 13-like	Down	Up
callose synthase 2-like	Down	Up
dehydrin Xero 1-like	Up	Down
DNA damage-binding protein 1a	Down	Up
exocyst complex component EXO70B1-like	Up	Down
Glu S.griseus protease inhibitor-like	Up	Down
lachrymatory-factor synthase-like	Down	Up
mannosyl-oligosaccharide glucosidase GCS1	Up	Down
probable (S)-N-methylcoclaurine 3′-hydroxylase isozyme 2	Up	Down
probable low-specificity l-threonine aldolase 1 isoform X2	Up	Down
probable prolyl 4-hydroxylase 7	Up	Down
protein disulfide isomerase pTAC5, chloroplastic	Down	Up
protein DJ-1 homolog B	Up	Down
protein KTI12 homolog isoform X2	Up	Down
protein SRG1-like	Up	Down
riboflavin biosynthesis protein PYRD, chloroplastic	Up	Down
RNA-binding protein CP31B, chloroplastic-like	Down	Up
secoisolariciresinol dehydrogenase-like	Up	Down
small heat shock protein, chloroplastic-like	Down	Up
universal stress protein PHOS32-like	Up	Down
vesicle transport v-SNARE 13-like	Up	Down
vesicle-associated protein 2-1	Up	Down

## Supplementary Materials

Supplementary materials can be found at https://www.mdpi.com/1422-0067/21/22/8525/s1.
